# *In-situ*/*operando* study of Cu-based nanocatalysts for CO_2_ electroreduction using electrochemical liquid cell TEM

**DOI:** 10.3389/fchem.2025.1525245

**Published:** 2025-01-30

**Authors:** Jiawei Wan, Qiubo Zhang, Ershuai Liu, Yi Chen, Jiana Zheng, Amy Ren, Walter S. Drisdell, Haimei Zheng

**Affiliations:** ^1^ Materials Sciences Division, Lawrence Berkeley National Laboratory, Berkeley, CA, United States; ^2^ Department of Materials Science and Engineering, University of California, Berkeley, Berkeley, CA, United States; ^3^ Chemical Sciences Division, Lawrence Berkeley National Laboratory, Berkeley, CA, United States; ^4^ Liquid Sunlight Alliance, Lawrence Berkeley National Laboratory, Berkeley, CA, United States

**Keywords:** electrochemical liquid cell TEM, *in-situ*, *operando*, nanocatalysts, Cu-based catalysts, CO_2_ electroreduction, nanocatalyst restructuring

## Abstract

The structure of a nanocatalyst during electrocatalytic reactions often deviates from its pristine structure due to intrinsic properties, or physical and chemical adsorption at the catalytic surfaces. Taking Cu-based catalysts for CO_2_ electroreduction reactions (CO_2_RR) as an example, they often experience segregation, leaching, and alloying during reactions. With the recent breakthrough development of high-resolution polymer electrochemical liquid cells, *in-situ* electrochemical liquid cell transmission electron microscopy (EC-TEM) alongside other advanced microscopy techniques, has become a powerful platform for revealing electrocatalysts restructuring at the atomic level. Considering the complex reactions involving electrified solid-liquid interfaces and catalyst structural evolution with intermediates, systematic studies with multimodal approaches are crucial. In this article, we demonstrate a research protocol for the study of electrocatalysts structural evolution during reactions using the *in-situ* EC-TEM platform. Using Cu and CuAg nanowire catalysts for CO_2_RR as model systems, we describe the experimental procedures and findings. We highlight the platform’s crucial role in elucidating atomic-scale pathways of nanocatalyst restructuring and identifying catalytic active sites, as well as avoiding potential artifacts to ensure unbiased conclusions. Using the multimodal characterization toolbox, we provide the opportunity to correlate the structure of a working catalyst with its performance. Finally, we discuss advancements as well as the remaining gap in elucidating the structural-performance relationship of working catalysts. We expect this article will assist in establishing guidelines for future investigations of complex electrochemical reactions, such as CO₂RR and other catalytic processes, using the *in-situ* EC-TEM platform.

## 1 Introduction

The electrocatalytic CO_2_ reduction reaction (CO_2_RR) has attracted great attention as a carbon-neutral route for the large-scale transformation of CO_2_ into valuable chemicals and fuels (Birdja et al., 2019; Nitopi et al., 2019; Ma et al., 2020). Cu has been the only metal electrocatalyst known to produce multicarbon (C_2+_) products, such as ethanol, ethylene, and n-propanol at relatively high Faradic efficiencies (FEs), but is limited by low selectivity due to production of other products, such as CO, HCOO-, and H_2_ at fairly high FEs (Kuhl et al., 2012; White et al., 2015). The carbon-carbon (C-C) coupling between CO*-CO*, CO*-CHO*, or CO*-COH* intermediates is a fundamental rate-determining step in CO_2_RR for C_2+_ conversion (Zhang et al., 2023). Improving the C-C coupling beyond the activity limitations imposed by the scaling relation would be key to achieving enhanced CO_2_RR performance (Zhu et al., 2018; Jia et al., 2022; Li and Zhang, 2023). Forming multicomponent Cu-based catalysts has been considered an effective route to improve this C-C coupling process, as various bimetallic Cu-based catalysts have been developed to achieve improved CO_2_RR performance compared to the monolithic Cu catalysts (Zhu et al., 2018; Jia et al., 2022; Li and Zhang, 2023).

Many factors may contribute to the unique and emergent properties of Cu-based bimetallic electrocatalysts, including electronic, geometric, tandem effects, as well as the synergies of different effects (Li and Zhang, 2023). For instance, the hybridization of the atomic orbitals can shift the d-band center with respect to the Fermi level in a non-intuitive way. Such electronic effects of bimetallic electrocatalysts may profoundly impact the catalytic properties (Zhu et al., 2018). Additionally, the structure of a working electrocatalyst often deviates from its pristine design. For example, the Cu-based nanocatalysts often experience structural segregation, leaching, or alloying during CO_2_RR (Xie et al., 2022; Yang et al., 2022; Cao et al., 2023). The reactions at the surfaces, including dynamic oxidization-reduction and adsorption-desorption of intermediates, can lead to structural and chemical changes of the nanocatalyst (Liu et al., 2021; Kong et al., 2022; Hao et al., 2023; Sun et al., 2024). Thus, unveiling the restructuring dynamics of the Cu-based nanocatalysts during reactions and resolving the active catalytic sites are crucial for the design of electrocatalysts with enhanced CO_2_RR performance.

Recently, a variety of *in-situ/operando* characterization techniques have played a central role in monitoring the reconstruction of electrocatalysts under realistic reaction conditions. Spectroscopic X-ray methods such as X-ray diffraction (XRD) (Wang et al., 2017) and X-ray absorption spectroscopy (XAS) (Lin et al., 2020) have been employed to monitor the ensemble structural evolution of nanocatalysts during reactions; however, they do not provide detailed information about individual nanoparticles. Other advanced *in-situ/operando* spectroscopic techniques, such as Raman spectroscopy (Deng et al., 2016) and infrared (IR) spectroscopy (Katayama et al., 2018), provide chemical bonding evolution occurring on the surface of catalysts; however, they are unable to reveal detailed or atomic-level structures. While *in-situ* microscopy like scanning tunneling microscopy (STM) (Cao et al., 2018) allows us to reveal the atomic-scale structure of flat surfaces, it is difficult to resolve the fast structural changes of a nanocatalyst during reactions. Among them, *in-situ* electrochemical liquid cell transmission electron microscopy (EC-TEM) has demonstrated its versatility in visualizing the microscopic and spectroscopic changes of nanocatalysts during reactions in electrolyte with high spatiotemporal resolution (Yang et al., 2023; Wan et al., 2024; Zhang et al., 2024). *In-situ* EC-TEM is the only technique with the necessary spatial and temporal resolution for direct observation of nanocatalysts restructuring at the atomic level, as well as other materials transformations by atomic transport/diffusion (Eswaramoorthy et al., 2007; Chou et al., 2008; Kim et al., 2008; Ross, 2015; Smeets et al., 2015).

Directly probing the atomic structure dynamics of working electrocatalysts remains very challenging due to technical obstacles as well as the complex reaction environment at the solid-liquid interfaces. With the recent developments of polymer electrochemical liquid cells, direct observation of atomic dynamics of the electrified Cu catalyst surfaces during CO_2_RR has been achieved for the first time. This opens unprecedented opportunities to probe restructuring of various electrocatalysts during reactions with high-resolution using advanced *in-situ* EC-TEM.

In this article, we will first introduce the *in-situ* EC-TEM platform, including the development of electrochemical liquid cells and advanced microscopy techniques, which enables the *in-situ/operando* study of Cu-based nanocatalysts for CO_2_RR at the atomic level. Then, we will describe the protocol we developed for using *in-situ* EC-TEM to study CO_2_RR electrocatalysts. Besides the *in-situ* EC-TEM experiments, a series of complementary studies such as *operando* XAS, systematic control experiments, and cryo-EM study of intermediates are included. They form a multimodal characterization toolbox essential for systematically uncovering the atomic pathways of nanocatalyst restructuring and identifying active sites. This approach is critical for discerning potential artifacts and minimizing the risk of biased conclusions. Using Cu and CuAg nanowire catalysts as model systems, we will briefly show the results of our studies by using the *in-situ* EC-TEM platform and following the discussed protocols. Finally, we discuss the advancements, as well as the remaining gaps, in correlating the structural changes of a working catalyst with the performance. We expect this article will help establish guidelines for future study of complex reactions, such as CO_2_RR and other electrocatalytic reactions, using the *in-situ* EC-TEM platform.

## 2 Materials and methods

### 2.1 The *in-situ* EC-TEM platform

**FIGURE 1:**
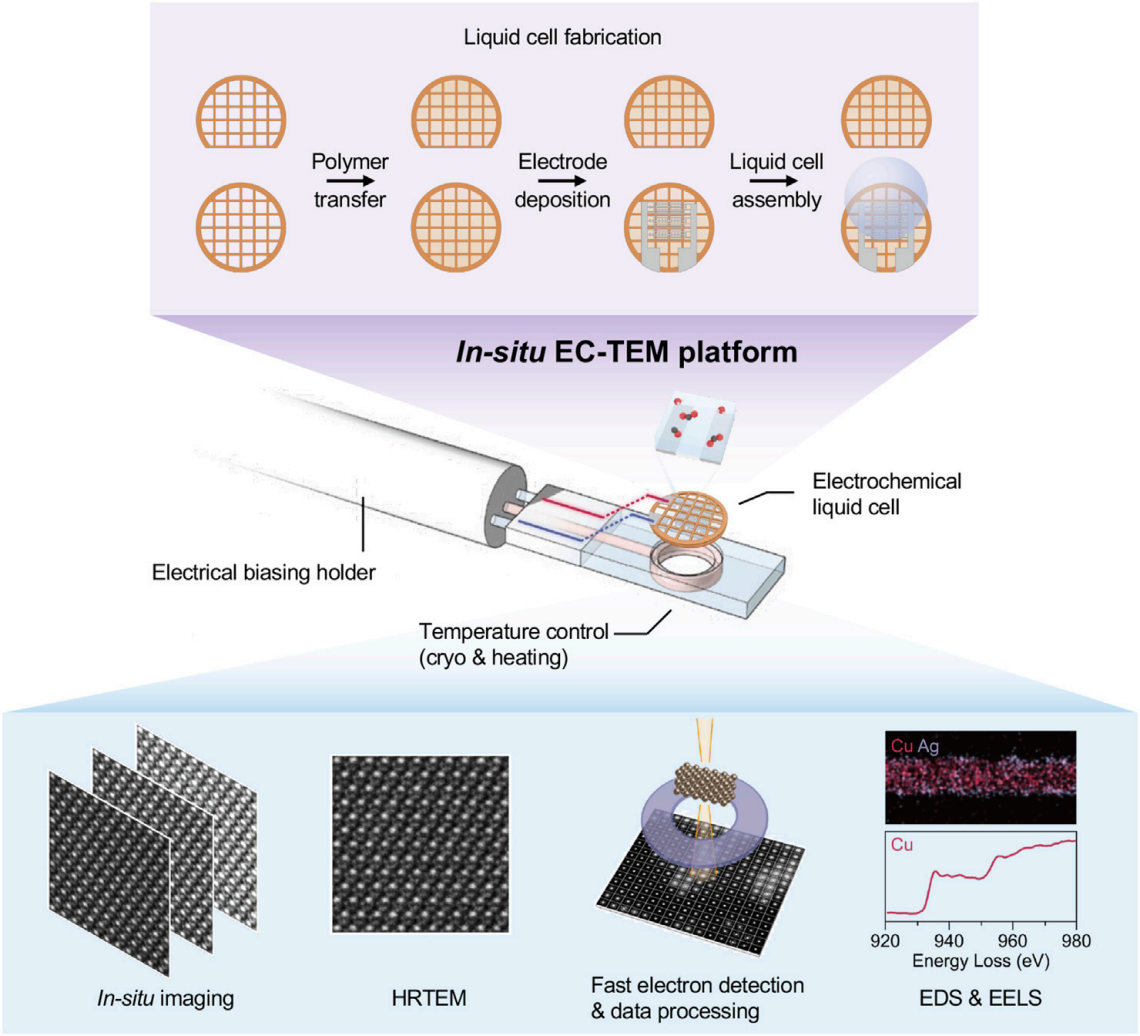
An *in-situ* electrochemical liquid cell TEM platform.

As shown in Figure 1, the electrochemical liquid cells allow examining samples in liquids under electrical biasing (through a sample stage) in the high-vacuum TEM. This forms a powerful *in-situ* EC-TEM platform, where various working nanocatalysts can be imaged with high spatiotemporal resolution under well-controlled electrochemical potentials and high-quality data sets can be collected. Nowadays, using a modern aberration-corrected electron microscope equipped with advanced detectors for imaging and analytical measurements, high-resolution images (movies or single-shot), energy dispersive X-ray spectroscopy (EDS), electron energy loss spectroscopy (EELS), and four-dimensional scanning transmission electron microscopy (4D-STEM) data can be routinely obtained. The key enablers for the advanced *in-situ* EC-TEM platform are the high-resolution electrochemical cells and a set of advanced microscopy methods designed for studying complex systems and reactions (e.g., CO_2_RR, or other electrocatalytic reactions).

The recent breakthrough of polymer electrochemical cells allows one to reveal the atomic dynamics of Cu catalyst surface restructuring during CO_2_RR. This polymer electrochemical liquid cell design enables high-resolution imaging and chemical analysis, such as EDS and EELS, to resolve the chemical composition and bonding structures of catalysts, which has been a challenging issue for the liquid cells with thick membrane. In addition, the polymer electrochemical liquid cells allow direct sample freezing at liquid nitrogen temperatures without breaking the membrane. Thus, the intermediates during electrocatalytic reactions can be carefully examined. The polymer electrochemical liquid cell meets all required conditions for studying electrocatalytic reactions. It offers unwavering advantages in revealing the dynamic structural changes of electrocatalysts during reactions at the atomic level compared with other methods.

The polymer electrochemical liquid cells with the two-electrode system allows for measurement of electrocatalytic reactions. Since its counter-electrode potential may drift over the course of the experiment, the three-electrode system is often desired for more accurate and stable electrochemical potential measurements. However, with systematic control experiments, the potential drift issue with the two-electrode system may be overcome. They may still be preferred, especially considering that fabrication of two-electrode electrochemical liquid cells is more cost and labor effective.

Cutting-edge microscopy techniques, as well as fast electron detection, can be applied to the *in-situ* EC-TEM platform to unveil the structural dynamics of working catalysts. The *in-situ* EC-TEM platform benefits from the rapid development of fast electron detection technology. Nowadays, capturing dynamic structural changes at 200 frames per second can be achieved routinely using a modern camera for *in-situ* TEM. The direct electron detection (DED) is capable of 11 microsecond readout times, extending the counting regime to higher doses compatible with *in-situ* TEM (Johnson et al., 2018). Although summing 10-100 images will be necessary to enhance the signal-to-noise ratio to produce interpretable images, it will also allow for investigating sub-nanometer-scale dynamics at previously unreachable speeds and possibly with reduced dose.

The fast imaging from *in-situ* TEM experiments produces large data, which requires advanced computer-assisted analysis. Automated imaging analysis with custom algorithms (drift correction, tracking, segmentation, etc.) can be directly applied to rapidly analyze the large data generated.

It is important to consider that nanocatalysts can be damaged by electron beam irradiation, especially under strong electron beam density for atomic-resolution imaging. It is well-known that electron beam damage is a major limitation affecting *in-situ* investigations, in which one seeks to minimize beam damage while simultaneously increasing the dose to achieve atomic resolution imaging at a fundamental level.

Methods have been developed to reduce beam damage. Low dose imaging has been well recognized for imaging many beam-sensitive materials, such as those in Li ion batteries, metal-organic frameworks, and zeolites (Esken et al., 2010). The use of liquid nitrogen cooling can also reduce the ionization and heating damage of the electron beam (Wiktor et al., 2012). Low dose imaging has been widely implemented in cryo-EM for biological samples, where individual objects can only survive being irradiated up to 10-20 e^−^/Å^2^ (Frank, 2002), Another feasible method is to introduce direct-detection electron-counting detectors for TEM imaging (Zhu et al., 2017; Zhang et al., 2018). Additionally, by reducing the energy of the electron beam (i.e., below 80 kV), knock-on damage can be prevented (Hashimoto et al., 2004).

### 2.2 A protocol using the multimodal characterization toolbox

**FIGURE 2:**
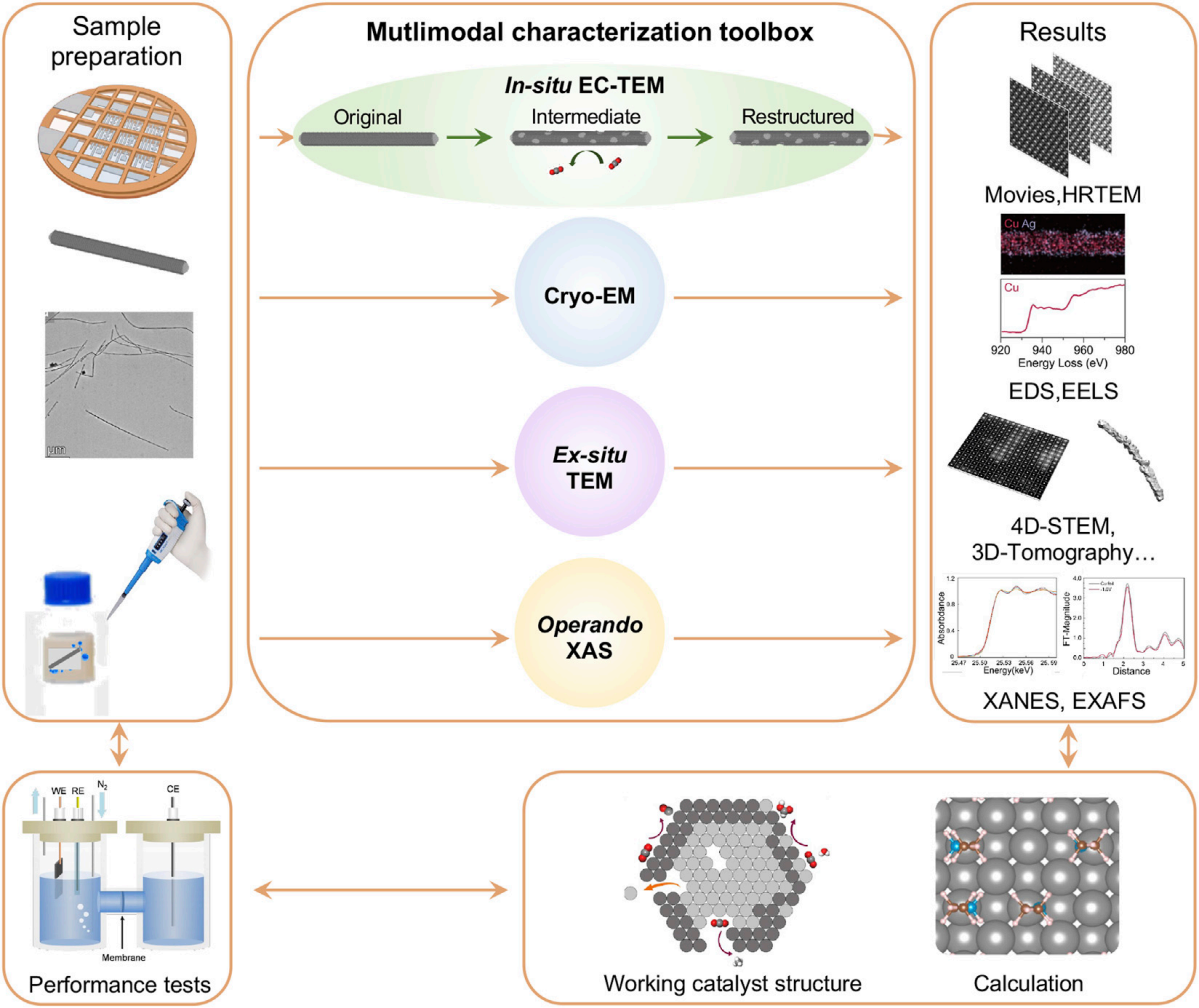
A protocol for *in-situ*/*operando* study of Cu-based nanocatalysts for CO_2_ electroreduction using electrochemical liquid cell TEM platform. A multimodal characterization toolbox is introduced for this study.

The *in-situ* EC-TEM platform provides great opportunities to directly observe and analyze electrochemical reactions in real-time at the atomic level. Besides CO_2_RR, it can also be applied to other electrocatalytic reaction systems, such as the hydrogen evolution reaction (HER), oxygen evolution reaction (OER), and oxygen reduction reaction (ORR). Although *in-situ* EC-TEM provides real-time insights into the structural and morphological evolution of catalysts, it is difficult to achieve an in-depth understanding of the corresponding catalytic mechanisms and build an overall realization of the real active species through a sole *in-situ* approach. Therefore, we designed a protocol using the multimodal characterization toolbox for comprehensive studies of electrocatalysis mechanisms, from sample preparation to advance *in-situ* and *ex-situ* microscopic and spectroscopic characterization, electrocatalytic performance testing, and theoretical calculation (Figure 2).

Sample preparation is the initial and critical step, playing a pivotal role in the success of the study. Different procedures are required to accommodate the specific requirements of different measurements. In a typical *in-situ* EC-TEM experiment, the as-synthesized nanocatalysts are dispersed in a liquid medium (e.g., ethanol, isopropanol, or others) and then drop-cast onto the electrodes of the electrochemical liquid cell bottom chip. After drying out the solution, a droplet of electrolyte (e.g., 1 μL of 0.1M KHCO_3_ solution saturated with CO_2_ gas) is added on the bottom chip, followed by covering with the top chip for further measurements. The liquid sample is sealed inside the liquid cell by Van der Waals forces between the top and bottom chips. It can be challenging to seal the liquid electrolyte inside the liquid cell due to drying or leaking; therefore, careful procedures and precautions are required to ensure successful experiments.

The *in-situ* EC-TEM enables the observation of morphological and local structural changes of individual working catalysts through the application of *in-situ* imaging, HRTEM, EDS, and EELS. Other microscopic and spectroscopic methods need to be conducted in parallel. For example, the intermediate structures of a catalyst during electrocatalytic reactions can be complex and fast-evolving, which may require more detailed analysis. Our newly-developed polymer electrochemical liquid cells allow fast freezing of the sample to cryogenic temperatures without breaking the membrane, which provides cryo-EM studies of the intermediates. Additionally, cryogenic transmission electron tomography (cryo-ET) will be used to characterize the catalysts after the catalytic performance test. Cryo-EM has been revolutionized by the introduction of DEDs due to their improved sensitivity (Brilot et al., 2012; Nogales, 2016). The highly sensitive electron detectors can acquire high-resolution TEM images under a sufficiently low electron dose condition and capture the structures before electron beam-induced damage occurs.

*Ex-situ* TEM measurements are useful to assist the interpretation of *in-situ* EC-TEM results. For example, electrocatalytic experiments can be carried out using a liquid cell or in bulk solution outside of the microscope. The structures of the catalysts can be examined by terminating the reactions at different potentials, and the results can be compared with the *in-situ* TEM observations.

As *in-situ* EC-TEM usually provides dynamic information of a single nanoparticle which may not reflect the overall evolution of catalysts under working conditions, *operando* X-ray spectroscopic techniques can be vital for the study of ensemble catalysts. For example, *operando* XAS unveils the valence state evolution and atomic coordination environment of catalysts in ensemble from X-ray absorption near-edge structure (XANES) and extended X-ray absorption fine structure (EXAFS) results. We can also employ the high energy resolution fluorescence detected (HERFD-) XAS, which has increased energy resolution that increases sensitivity to small changes in XANES pre-edge features.

Lastly, in addition to the methods discussed above, other surface-sensitive characterization techniques are also considered. For instance, Raman spectroscopy can provide valuable insights into the bonding information of intermediates, while X-ray photoelectron spectroscopy (XPS) is useful for probing the chemical and electronic properties of catalytic materials. It is worth noting, however, it presents significant challenges using *in-situ* XPS presents to study electrochemical processes in liquid environments, and further technical innovations are required to overcome these limitations.

To establish the correlation between the observed catalyst structural dynamics and electrocatalytic performance, it’s important to conduct electrolysis of designed catalysts. Take CO_2_RR as an example: testing in both H-type cell and membrane electrode assembly (MEA) is preferred for both laboratorial research and industrial applications. Moreover, the mortem measurements from the multimodal characterization toolbox can further verify the comparability with *in-situ* experiments for a thorough study on the structure-performance correlation of electrocatalysts under working conditions. Finally, theoretical calculations will provide atomistic insights into reaction mechanisms and pathways, as well as active sites. Coupled with complementary experimental observations, it helps interpret complex behaviors and guide the design of more efficient and selective catalysts.

## 3 Results

### 3.1 The study of Cu nanowire electrocatalysts

Cu nanowires are first used as the model catalysts for CO_2_RR in CO_2_-saturated 0.1 M KHCO_3_ electrolyte and the *in-situ* EC-TEM platform is applied to study the structural transformations. Under CO_2_RR conditions, we monitor the interfaces between the Cu catalyst and the electrolyte to reveal structural changes at the atomic level.

**FIGURE 3:**
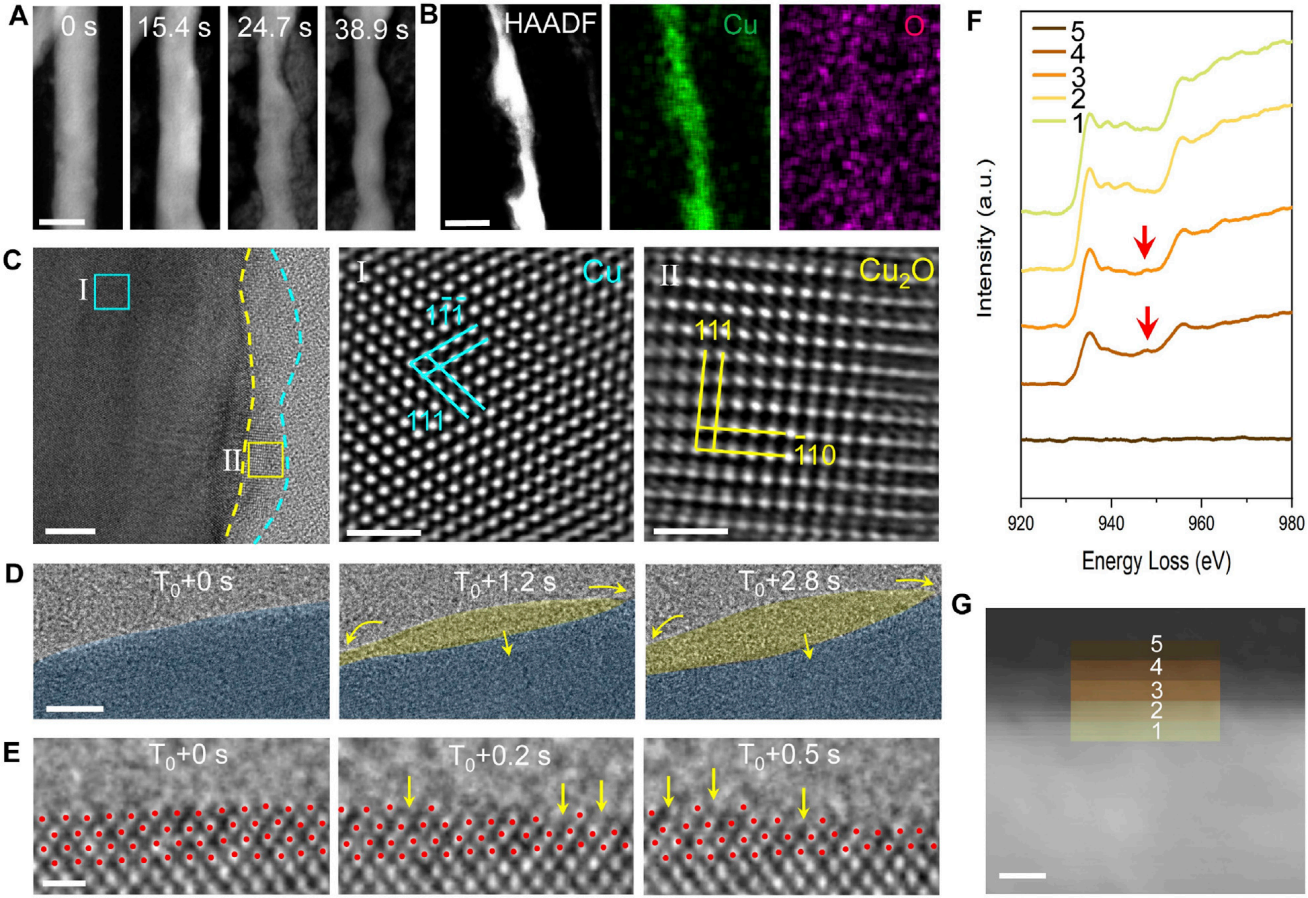
TEM analysis of the dynamic structural evolution of Cu catalysts during CO₂ reduction. **(A)** *In-situ* HAADF images illustrate morphological changes in the Cu catalyst during the CO₂ reduction reaction. Scale bar: 50 nm. **(B)** EDS maps display the elemental distribution within the Cu catalyst post-reaction. Scale bar: 20 nm. **(C)** HRTEM image reveals an oxide coating layer present on the Cu catalyst after CO₂ reduction. Scale bar: 5 nm, 1 nm, 1 nm. **(D)** An *in-situ* HRTEM image sequence captures the formation and fluctuation of an amorphous interphase. Scale bar: 1 nm. **(E)** Atomic-resolution imaging provides detailed insights into the transformation of crystalline Cu into the amorphous interphase. Scale bar: 5 Å. **(F)** EELS spectra analysis of distinct regions at the interface between crystalline Cu and the amorphous interphase reveals that the amorphous region is rich in Cu⁺ ions, while the crystalline section is predominantly zero-valent Cu. **(G)** HAADF image of the amorphous-crystalline interface in an activated Cu nanowire, with colored squares marking areas where EELS spectra were obtained. Scale bar: 5 nm.

As shown in Figure 3A, Cu nanowire catalysts gradually dissolve into the electrolyte under the potential of −1.1 V vs. Pt counter electrode. Over time, the diameter of the Cu nanowires decreases, and the initially flat surfaces evolve with the appearance of pronounced curvatures. We anticipate that such a dissolution process compromises the stability and lifetime of the catalyst, although the increased surface curvature generates a high density of active sites that enhance catalytic activity. The EDS map of the reacted nanowires (Figure 3B) reveals a uniform distribution of Cu throughout the nanowire, while O levels between the nanowire and the background electrolyte remain similar. Therefore, the EDS analysis indicates that the nanowires are composed of pure copper, with no detectable oxidized surface layer. From here, one may conclude that the Cu catalyst remains in its metal crystalline state throughout the CO₂RR process while dissolution occurs at the catalyst surfaces. However, further careful studies reveal differently.

By incorporating cryo-EM techniques, we obtain the native state of Cu during the reaction by fast-freezing the working catalyst. As shown in Figure 3C, a thin amorphous layer is present on the Cu surface. To rule out the influence of electron beam irradiation, we conduct *ex-situ* control experiments. The results confirm the generation of amorphous structures is intrinsic under electrochemical conditions. For example, we found electron-beam irradiation promotes crystallization of this amorphous interphase, resulting in the formation of Cu_2_O nanocrystals (Zhang et al., 2024). Therefore, we conclude that an amorphous structure on the catalyst surfaces generated by a working Cu is not due to electron beam damage.

The HRTEM image sequence (Figure 3D) from *in-situ* TEM captures the dynamics of the amorphous interphase. At 0 s, the surface of the crystalline Cu appears clean. After approximately 1 s, an amorphous layer forms and grows into the crystalline Cu, as highlighted by the yellow arrows. The changes in the projected area of the two phases over time show clear opposing trends, highlighting the predominant interconversion behavior. Atomic-resolution dynamic imaging (Figure 3E) reveals that the appearance of an amorphous interphase alters the vacancy formation energy of copper atoms at the interface between crystalline Cu and amorphous region, reducing the energy difference between atomic steps and terraces. As a result, Cu atoms readily dissolve at atom steps and on atom terraces, leaving some atomic pits as pointed by the yellow arrows.

We conduct EELS measurements to examine the valence state of the amorphous region as well as the crystalline Cu using cryo-EM techniques. As shown in Figures 3F, G, only Cu^0^ signals are detected within the crystalline Cu region, whereas in the amorphous region, a peak corresponding to Cu⁺ ions emerges and intensifies with distance from the interface. This result confirms the presence of Cu⁺ ions within the amorphous interphase. Thus, by combining the *in-situ* TEM platform with advanced polymer electrochemical liquid cells, we successfully overcome the challenges and limitations of prior *in situ* techniques and uncover phenomena previously inaccessible.

Our direct observations indicate that the amorphous interphase can mediate surface reconstruction, resulting in a stepped surface that enhances catalytic performance. Theoretical calculations suggest that the performance improvement may be due to the formation of stepped surfaces and the coexistence of Cu^0^ and Cu^1^⁺ ions within the amorphous interphase. The discovery of the amorphous interphase brings a new perspective to catalyst research and the design of advanced catalysts.

### 3.2 The study of CuAg nanowire electrocatalysts

CuAg bimetallic nanocatalysts have been developed to improve the conversion efficiency of CO_2_ to C_2+_ products. Some studies show the localized CO produced by Ag is key towards efficient C_2+_ production with subsequent C-C bonding on Cu (Chen et al., 2020; Zhang Y. et al., 2024). Others consider CuAg alloy to be beneficial in increasing the binding energy of the intermediates during CO_2_RR, improving the FE_C2+_ (Li Y. C. et al., 2019). As Cu-based catalysts often experience dynamic restructuring during electrocatalytic reactions, fundamental questions have emerged for understanding their structure-performance correlations. First, the structural evolution mechanism of CuAg nanocatalysts during CO_2_RR remains elusive, which impedes the knowledge of enhanced catalytic performance. Second, the identification of active sites of working catalysts remains lacking. For example, the valence state of active sites under working conditions is still under debate. In addition, the interaction between Ag and Cu and its influence on the catalytic reaction pathways are also not well understood. The major hurdle is the inability to directly monitor the structural evolution of electrocatalysts under operating conditions due to the fast, complex dynamic transformation behaviors during CO_2_RR.

**FIGURE 4:**
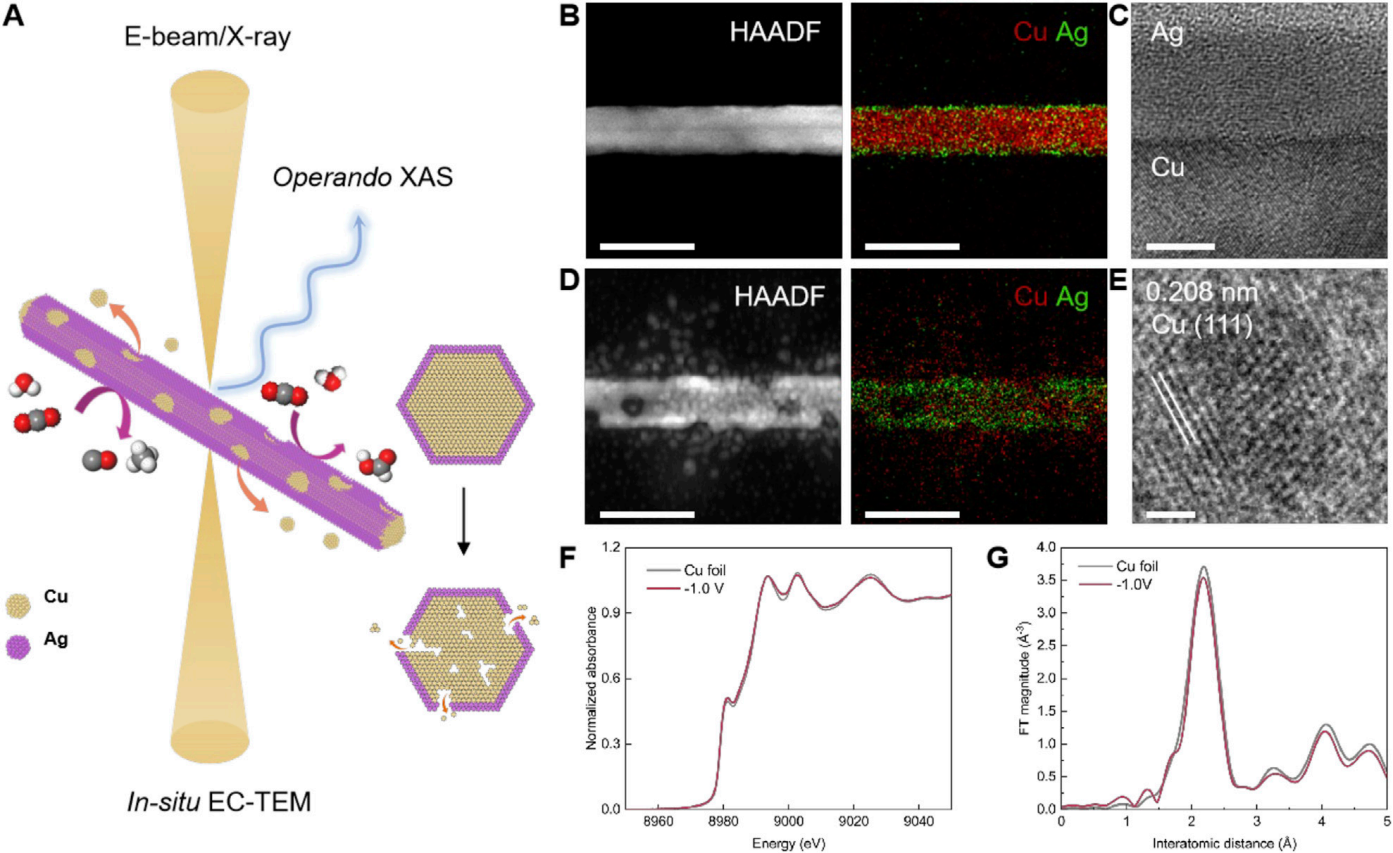
Observing the structural dynamics of CuAg nanowires during CO₂RR via the multimodal characterization toolbox. **(A)** Schematic illustration of the multimodal toolbox employed to track the dynamic behaviors of CuAg nanowires under CO_2_RR conditions. **(B)** HAADF-STEM and EDS mapping of CuAg nanowires before CO_2_RR. Scale bar: 50 nm. **(C)** HRTEM image shows the Cu-Ag interface in CuAg nanowires. Scale bar: 5 nm. **(D)** HAADF-STEM and EDS mapping of CuAg nanowires during CO_2_RR at −1.0 V vs. RHE. Scale bar: 50 nm. **(E)** HRTEM image shows the exposure of the Cu (111) facet on CuAg nanowires surface after electrocatalysis. Scale bar: 1 nm. **(F)** XANES and **(G)** FT-EXAFS spectra of Cu K-edge for Cu foil and CuAg nanowires at −1.0 V vs. RHE, respectively.

A multimodal approach coupling *in-situ* EC-TEM and *operando* time-resolved XAS with high spatiotemporal resolution is crucial to study CuAg and other electrocatalysts to gain mechanistic insights and enable improved control of catalyst structure and performance (Figure 4A). *In-situ* EC-TEM uncovers the morphological and local structural changes of individual working catalysts, while *operando* time-resolved XAS unveils the valence state evolution and atomic coordination environment of catalysts in ensemble.

We synthesize CuAg nanowire as the model system. Cu nanowires are first prepared using a hydrothermal method, followed by Ag deposition through the heterogeneous nucleation in liquid. The HAADF-STEM image presents the typical morphology of a CuAg nanowire (Figure 4B). EDS mapping results further show the elemental distribution of Cu and Ag. The Cu nanowire is covered with an ultrathin Ag layer, forming a core-shell configuration. HRTEM image also confirms the atomic crystalline structure of the Cu core and Ag shell (Figure 4C). TEM characterizations of the CuAg nanowires are carried out after CO_2_RR at −1.0 V vs. RHE. HAADF-STEM and EDS mapping show the nanowires display surface roughening, exhibiting granular Cu nanoparticles generated from the as-prepared CuAg nanowire, resulting in the formation of voids and cracks inside the nanowire (Figure 4D). HRTEM also displays the atomic structure of a reconstructed Cu nanoparticle (Figure 4E). However, compared with Cu, Ag is relatively stable without obvious change of distribution.

*Operando* time-resolved XAS results show that the metallic Cu phase is maintained during electrocatalytic conditions. The XANES of the Cu K-edge of the CuAg nanowires catalysts match the corresponding reference of Cu foil (Figure 4F). FT-EXAFS reveals the characteristic peaks at 2.2 Å, which correspond to the Cu-Cu scattering paths in the metallic Cu spectra (Figure 4G).

In conclusion, by combing multimodal characterizations using *in-situ* EC-TEM and *operando* XAS, we are able to track catalyst structure and valence state evolution. It establishes a solution towards resolving the complex and dynamic nature of electrocatalysts during reactions and is crucial for understanding their structure-performance correlations.

## 4 Discussion

We have demonstrated a research protocol for studies of electrocatalytic mechanisms, aiming to reveal structural evolution of electrocatalysts during reactions through an *in-situ* EC-TEM platform. This powerful platform, which utilizes the recent breakthrough development of high-resolution polymer electrochemical liquid cells, enables the capture of atomic-scale structural dynamics of working catalysts, allowing us to correlate catalyst structure with performance. To gain a comprehensive understanding, systematic studies using the multimodal characterization toolbox approaches are essential. By employing *in-situ* and *ex-situ* advanced microscopic and spectroscopic techniques to examine working electrocatalysts, we achieve a holistic view of the structural and valence states evolution of active catalysts.

Using the Cu nanocatalyst as a model system, we showcase applying *in-situ* EC-TEM to study complex systems and reactions that were previously unreachable. The atomic-scale dynamics of Cu catalyst surfaces reveal the importance of visual and microscopic studies to elucidate the underlying mechanisms of the interfacial amorphization for Cu catalysts. In addition, by employing CuAg nanocatalysts as the model systems for CO_2_RR, we show how multiscale complementary characterizations can significantly enhance our insights.

It is important to highlight the necessity of conducting electrolysis measurements on designed catalysts to establish a clear correlation between the observed structural dynamics and electrocatalytic performance. The H-type cell is commonly used in laboratory research for investigating reaction mechanisms and evaluating catalyst performance. MEA is generally preferred for their compact design, high current density capabilities, and efficient mass and ion transport. Unlike H-type cell, MEA closely replicates the conditions of commercial electrolyzers, making them more suitable for evaluating catalysts under practical operating conditions.

## 5 Conclusion

In summary, the polymer electrochemical liquid cell TEM offers superb high spatial resolution, and it allows chemical analysis with EDS and EELS, as well as other advanced microscopy techniques. It forms a powerful platform for *in-situ* characterization of electrocatalysts during CO_2_RR. We have demonstrated a multimodal characterization toolbox by combining the *in-situ* high-resolution EC-TEM with various advanced electron microscopy techniques and *operando* X-ray spectroscopy that can be applied uniquely and effectively to reveal the structural evolution of electrocatalysts correlated with their performance. It is also noted that the current *in-situ* EC-TEM has limitations, for example, in detecting the adsorbates and the reaction products. Thus, complementary studies using XPS, Raman and other spectroscopy methods can be helpful and necessary. This work highlights the future opportunities to advance the multimodal characterization of catalysts during CO_2_ electroreductions and other electrocatalytic processes.

## Data Availability

The original contributions presented in the study are included in the article/supplementary material, further inquiries can be directed to the corresponding author.
